# Characterization of recombinant human and bovine thyroid-stimulating hormone preparations by mass spectrometry and determination of their endotoxin content

**DOI:** 10.1186/1746-6148-9-141

**Published:** 2013-07-16

**Authors:** Sandra Schaefer, Paul O Hassa, Nadja S Sieber-Ruckstuhl, Marion Piechotta, Claudia E Reusch, Bernd Roschitzki, Felicitas S Boretti

**Affiliations:** 1 Clinic for Small Animal Internal Medicine Vetsuisse Faculty, University of Zurich, Zurich, Switzerland; 2 Institute of Veterinary Biochemistry and Molecular Biology, Vetsuisse Faculty, University of Zurich, Zurich, Switzerland; 3 Clinic for Cattle, Endocrinology Laboratory, University of Veterinary Medicine Hanover, Hanover, Germany; 4 Functional Genomics Center Zurich (FGCZ), University of Zurich/ETH Zurich, Zurich, Switzerland

**Keywords:** Bovine TSH, Recombinant human TSH, Mass spectrometry, Endotoxin

## Abstract

**Background:**

The TSH stimulation test to confirm canine hypothyroidism is commonly performed using a recombinant human TSH (rhTSH), as up to date, canine TSH is not yet commercially available. Limiting factors for the use of rhTSH are its high costs and occasional difficulties in product availability. Less expensive bovine TSH preparations (bTSH) purified from bovine pituitary glands are readily commercially available. The aim of this study was to evaluate two different bTSH products as alternative to rhTSH using mass spectrometry.

**Results:**

More than 50 proteins, including other pituitary hormones, bovine albumin, hemoglobin, and tissue proteins were identified in the bTSH preparations. In contrast, rhTSH proved to be a highly pure product. Significantly higher endotoxin levels could be detected in all bTSH products compared to the rhTSH.

**Conclusions:**

Both bTSH products are crude mixtures and therefore not an acceptable alternative to rhTSH. Their use should be discouraged to prevent unintended side effects.

## Background

The TSH stimulation test has long been recognized as an accurate examination of thyroid function for the diagnosis of hypothyroidism in dogs. Increases in T4 (thyroxin) after the administration of exogenous TSH provide an assessment of the functional reserve capacity of the thyroid gland, and this helps discriminate true hypothyroidism from other conditions with low T4 secretion [1,2]. Canine TSH would be the ideal substance to perform the test, and although canine recombinant TSH could successfully be synthesized, it is not yet commercially available [3,4]. Therefore, the test is commonly performed using a recombinant human TSH (rhTSH) preparation [5-8]. Limiting factors, however, are the high cost of rhTSH and the occasional difficulties in obtaining the product. TSH purified from bovine pituitary glands (bTSH), which is easily commercially available, was widely used before rhTSH had been introduced in veterinary medicine. Equivalent biological activity of a bTSH product and the rhTSH has been shown in healthy beagle dogs [9]. However, in veterinary but also human medicine side effects after the use of bTSH were occasionally observed including allergic and anaphylactoid reactions [10-14]. Although they were assumed to result from impurities contained in the bTSH, a detailed characterization of commercially available bTSH to confirm this assumption has never been performed.

Mass spectrometry (MS) has been used to monitor protein purifications and to identify components in protein or peptide mixtures [15-18]. MS has also been suggested as an excellent analytical tool to establish key differences between recombinant proteins and their natural counterparts, especially when these products are intended for clinical use [17]. To our knowledge, no MS studies have been conducted to describe the composition of commercial bTSH products.

Important contaminants found in parenteral drugs are endotoxins, also known as lipopolysaccharides (LPSs), components of the outer membrane of gram-negative bacteria. LPS in the blood circulation can lead to systemic inflammation and endotoxin shock [19]. Possible sources of endotoxins in drugs are chemicals, raw materials, or equipment used in the preparation and purification of the products. The Limulus Amebocyte Lysate (LAL) test is an officially accepted and very sensitive method to test endotoxin levels. To date, there are no published studies that compare the endotoxin content of different TSH preparations.

Knowledge of the composition of bTSH and the identification of potentially dangerous contaminants, e.g., endotoxins in this product are both prerequisites for veterinary clinical use of bTSH.

Therefore, aim of this study was to characterize commercially available purified bTSH (two different products) and rhTSH (one product) by MS. In addition, the levels of endotoxin were measured in both preparations by the Limulus endotoxin assay.

## Results

### Peptide and protein identification in bTSH

**Figure 1:**
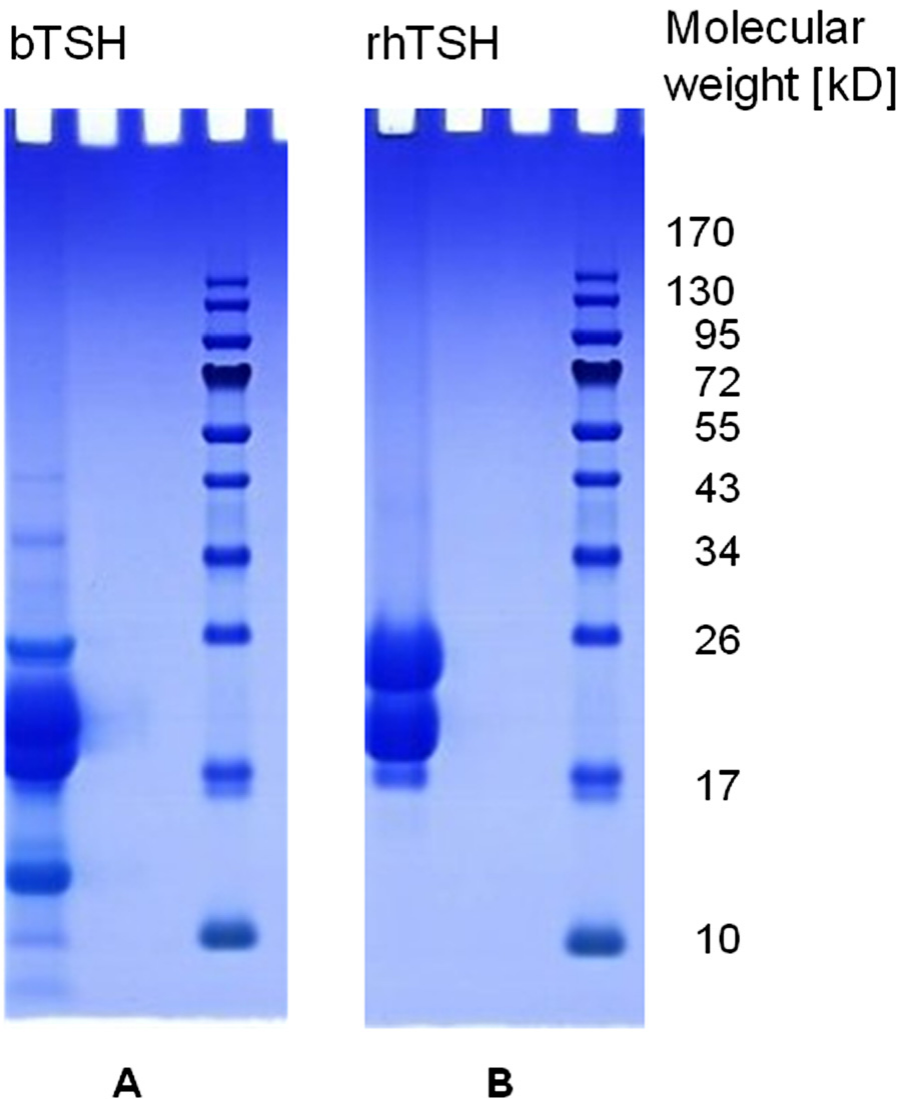
**Bis-Tris SDS PAGE gel of bTSH (product 1) and rhTSH (each 70 μg/lane); after electrophoretic separation, protein bands were detected by Coomassie Blue staining.** The right lane of each gel represents the molecular weight marker (Prestained Protein Ladder; Page Ruler, Fermentas; Thermo Scientific, Glen Burnie, MD, USA), the left lane of gel **A** and **B**, the bTSH and rhTSH, respectively.

bTSH and rhTSH samples were separated by SDS-PAGE before analyses by MS. Selected examples of Bis-Tris gels after electrophoresis and Coomassie Blue staining of bTSH and rhTSH-loaded gels are shown in Figure 1A (bTSH product 1, Sigma Aldrich) and 1B (rhTSH), respectively.

In addition to bTSH, more than 50 other proteins were identified in all bTSH samples, including other pituitary hormones such as bovine prolactin, LH, arginine vasopressin (AVP), proopiomelanocortin (POMC), GH, oxytocin-neurophysin, and glycoprotein hormone alpha chain. Moreover, bovine albumin, hemoglobin, and cytoskeleton proteins were found in many of the tested samples. Interestingly, major differences in the composition of albumin were observed among the different lots of bTSH (bTSH product 1, Sigma Aldrich), while the pituitary hormones (prolactin, LH, AVP, glycoprotein hormone alpha chain, and TSH) were not significantly different between the lots. A list of selected proteins of both preparations is presented in Tables 1 and 2, respectively. A complete summary of all identified proteins in bTSH product 1 (three different lots) and 2 (one lot) are presented as Additional file 1: Table S1 and Additional file 2: Table S2, respectively; accession number and number of assigned spectra are given.

**Table 1:** Selected proteins identified in the bTSH product 1 (Thyrotropic hormone from bovine pituitary, Sigma Aldrich; 3 lot numbers) by database search following mass spectrometry

**Identified proteins**	**LOT 069 K1588**	**LOT 119 K1583**	**LOT 040 M1246**
Prolactin	143	105	60
Lutropin subunit beta	100	84	86
Vasopressin-neurophysin2-copeptin	85	87	61
Thyrotropin subunit beta	76	64	44
Glycoprotein hormones alpha chain	57	73	75
Pro-opiomelanocortin	36	35	42
Serum albumin	25	1	0
Somatotropin	12	11	8
Beta-2-glycoprotein 1	5	2	0
Hemoglobin subunit beta	4	2	3
Oxytocin-neurophysin 1	4	2	2

Numbers of assigned spectra are given (corresponding to the numbers of the acquired spectra per protein).

**Table 2:** Selected proteins identified in the bTSH product 2 (TSH, bovine pituitary, Calbiochem Merck; 1 lot number) by database search following mass spectrometry

**Identified proteins**	**LOT**
	**D00106386**
Beta-2-glycoprotein 1	52
Serum albumin	51
Lactotransferrin	45
Hemoglobin subunit beta	32
Lutropin subunit beta	16
Glycoprotein hormones alpha chain	13
Thyrotropin subunit beta	13
Vasopressin-neurophysin 2-copeptin	7
Somatotropin	6
Pro-opiomelanocortin	4
Hemoglobin subunit alpha	3
Follitropin subunit beta	3

Numbers of assigned spectra are given (corresponding to the numbers of the acquired spectra per protein).

### Peptide and protein identification in rhTSH

In the rhTSH only TSH and several enzymes (e.g., caspase, galectin, serpin, calmodulin-like protein, and glyceraldehyde-3-phosphate dehydrogenase species) could be identified. In contrast to bTSH, the composition of rhTSH was extremely consistent between the different lot numbers.

A list of selected proteins from the two lot numbers of rhTSH is shown in Table 3 and is given as the number of assigned spectra. A complete summary of all identified proteins in the rthTSH products is presented as Additional file 3: Table S3; accession number and number of assigned spectra are given.

**Table 3:** Selected proteins identified in the rhTSH preparation (Thyrogen, Genzyme GmbH; 2 lot numbers) by database search following mass spectrometry

**Identified proteins**	**LOT**	**LOT**
	**A8035H40**	**A8063H19**
Thyrotropin subunit beta	116	126
Glycoprotein hormones alpha chain	25	26

Numbers of assigned spectra are given (corresponding to the numbers of the acquired spectra per protein).

### Estimation of pituitary protein level

**Figure 2:**
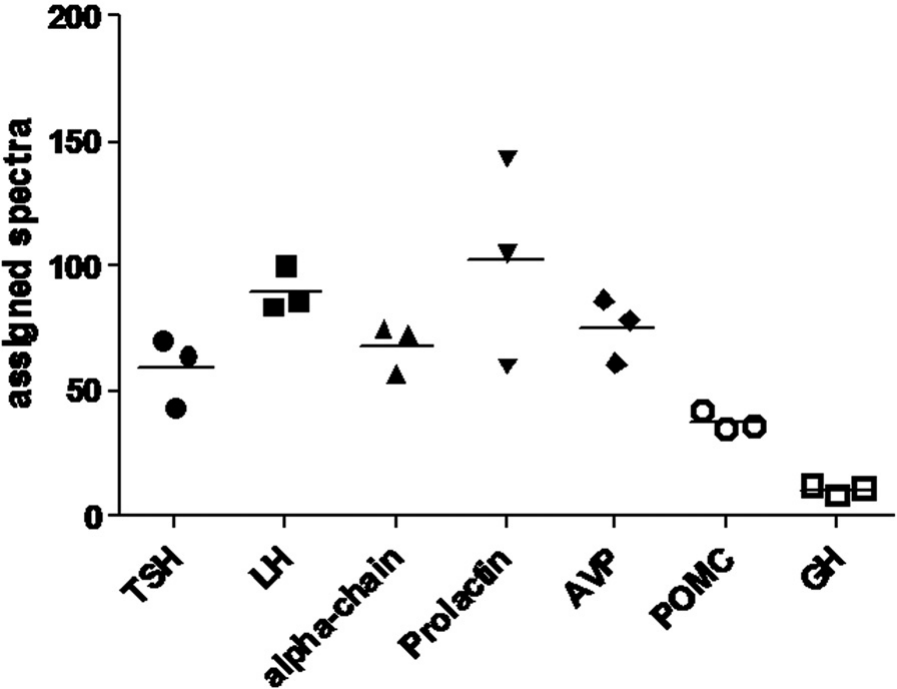
**Numbers of assigned spectra of the hormones detected in the bTSH (product 1, Sigma Aldrich).** Each symbol represents a lot number. The line represents the median of the 3 different lot numbers of each hormone. Thyroid stimulating hormone (TSH), luteinizing hormone (LH), arginine vasopressin (AVP), proopiomelanocortin (POMC), growth hormone (GH).

The number of detectable peptides of the identified pituitary proteins was comparable. Therefore it was assumed that the number of the detected spectra reflected the individual abundances of the proteins, which allowed an estimation of the hormone quantities. Based on this assumption, the most commonly occurring proteins in the bTSH product 1 (Sigma Aldrich) were prolactin and LH and although not statistically significant there was a trend towards higher levels of both hormones compared to the TSH (p = 0.1) and a trend towards lower GH levels compared to those of TSH, LH and prolactin (Figure 2). The rhTSH preparation had only minor amounts of proteins other than TSH.

Contaminants such as keratin or trypsin, and proteins that were detected in bTSH and in rhTSH were eliminated from the analyses.

### Measurement of LH and GH by ELISA

LH and GH were additionally measured by ELISA to confirm the findings of the MS with respect to the quantity of the hormone levels. The LH levels in the bTSH (product 1, Sigma Aldrich) samples were significantly higher compared to GH levels. Variations between the three different bTSH lots were only minimal and not statistically significant. Both, GH and LH levels in the rhTSH preparations were undetectable (Table 4).

**Table 4:** Bovine luteinizing hormone (LH) and bovine growth hormone (GH) concentrations in bTSH and rhTSH preparations as determined by ELISA

**TSH product**	**LOT**	**LH (μg/ml)**	**GH (μg/ml)**
**bTSH**	040 M1246	34.9	2.59
		37.4	2.29
	119 K1583	35.4	2.97
		34.5	2.75
**rhTSH**	A8063H19	< 1.2 ng/ml	< 0.8 ng/ml

### Endotoxin levels

Results of the LAL kinetic chromogenic assays of bTSH and rhTSH preparations at different dilutions are presented in Table 5. Endotoxin levels of all bTSH preparations were significantly higher compared to rhTSH. Further, large variations in endotoxin levels were observed among the different lots of the bTSH product 1 (Sigma Aldrich).

**Table 5:** Endotoxin concentrations in two bTSH products (Sigma Aldrich, 3 lot numbers; Calbiochem Merck 1 lot number) and rhTSH (2 lot numbers) as determined by Limulus amebocyte lysate (LAL) kinetic chromogenic assay

**Sample**	**LOT**	**Endotoxin/ml**	**Endotoxin/mg**
**bTSH**	069 K1588	434 EU/ml	80.4 EU/mg
(Sigma Aldrich)	119 K1583	158.4 EU/ml	29.3 EU/mg
	040 M1246	133.6 EU/ml	24.7 EU/mg
**bTSH**	D00106386	622 EU/ml	12.4 EU/mg
(Calbiochem Merck)			
**rhTSH**	A8035H40	0.15 EU/ml	0.14 EU/mg
(Genzyme GmbH)	A8063H19	<0.05 EU/ml	<0.04 EU/mg

## Discussion

Mass spectrometry analyses demonstrated that all bTSH samples of two different products contained in addition to TSH, more than 50 other proteins, including not only other pituitary hormones (e.g. LH, prolactin, AVP, POMC, and GH) but also bovine albumin, hemoglobin, and several tissue proteins (e.g., collagen, superoxide dismutase, and cathepsin). In contrast, in the rhTSH samples only TSH and a low number of enzymes used for its production were detected.

Recombinantly produced proteins usually contain only the protein or peptide coded by the target gene, and some minor enzymes used in the commercial production process. rhTSH (Thyrogen) is produced in genetically engineered cell lines (Chinese hamster ovary cells- CHO) and is highly purified by a combination of ion exchange and dye affinity chromatography [20] (Genzyme Corporation, http://www.genzyme.com).

In contrast to rhTSH, the bTSH products are purified proteins, prepared from the entire bovine pituitary gland. Depending on the purification procedure, it may be difficult to obtain a real pure product, particularly if proteins with high similarities to the target protein (in our case TSH) are present. It was therefore not surprising that other pituitary hormones were found in the various bTSH lots. Major cell types of the anterior pituitary are somatotropic cells, constituting approximately 50% of the cell population, followed by lactotropic (10-25%), corticotropic (10-20%), gonadotropic (10%), and thyrotropic cells (10%) [21].

Based on the high proportion of somatotropic cells in a pituitary mixture, a higher quantity of growth hormone would be expected if the protein purification method was non-specific. However, the proportion of GH in bTSH as determined by MS analyses and ELISA was low, confirming that the TSH purification was to some extent specific.

TSH belongs to the glycoprotein hormone family that includes FSH, hCG and LH; they are heterodimeric proteins and consist of a common α-subunit (or α-chain) and a unique β-subunit, which confers biological specificity to each hormone. Due to the very high homology between TSH and LH, separation of the two hormones is difficult and necessitates extensive purification procedures [22-24], which in turn is laborious and expensive. Based on our results showing a rather high proportion of LH (demonstrated by MS and ELISA), it can be assumed that such a special purfication procedure has not been applied in the case of our bTSH.

In a previous study, we found that the administration of 75 μg rhTSH to healthy beagle dogs resulted in stimulation of T4 similar to that caused by administration of 500 μg bTSH (product 1) [9]. Based on the current results, the more than six times higher dose of bTSH required to elicit the same effect as rhTSH can be explained by the high content of contaminating proteins in the bTSH lots. As mentioned above, the amounts of GH in the bTSH products were low, which led us to assume that at least some specific purification procedure was used; the more surprising was the finding of the highly variable amounts of bovine albumin that we could detect. Before rhTSH was available, bTSH given to human patients caused allergic reactions in up to 43% of the cases; some patients required emergency treatment [12-14]. Contaminating proteins such as albumin or globulins and antibodies against these proteins were suspected to be a likely cause and most side effects were observed after repeated injections of bTSH [12,13]. In veterinary medicine, side effects after the use of bTSH include allergic and anaphylactoid reactions [10,11]. Although these reactions were assumed to result from impurities contained in the bTSH, a detailed characterization to confirm this assumption has not been performed. If foreign albumin is administered, species differences in albumin can lead to anaphylactic and immunologic reactions. This has been shown both in human patients and in dogs [25-30]. Clinical symptoms in dogs receiving non-canine albumin ranged from edema (facial, distal limbs), urticaria, vomiting, and diarrhea to severe shock-like reactions (hypotension, collapse). Some of these symptoms have also been observed in dogs receiving bTSH, making adverse reactions to albumin contained in bTSH a likely factor. In one veterinary study, reactions had only been observed after repeated administration of bTSH. The authors hypothesized that these reactions were the result of hypersensitization [11]. Clearly, to avoid hypersensitization, a canine TSH would be the most appropriate substance for performing the TSH stimulating test in dogs. However, to our knowledge, purified canine TSH is not commercially available in sufficient amounts and although recombinant canine TSH could successfully be synthesized, it is not yet on the market [3,4].

However, in one report, two fatal outcomes were described after a single administration of bTSH to dogs that had no prior exposure to bTSH [10]. A possible explanation in these cases, in which a hypersensitization can be excluded, would be the high endotoxin concentration in some bTSH lots. Based on the LAL assay, the endotoxin concentration in bTSH was up to 500 times higher than that measured in rhTSH (434 and 0.27 EU/mL, respectively). Endotoxins in the blood circulation can lead to systemic inflammation and endotoxin shock with severe cardiovascular disturbances, multi-organ failure, and possible fatal outcome [19]. The highly variable endotoxin content in different lots makes adverse reactions unpredictable, and hence, the administration of bTSH to dogs is risky. Based on the high level of purity and the lack or minimal amount of endotoxin content, adverse reactions to rhTSH are unlikely. Therefore, compared to bTSH, rhTSH can be regarded as a much safer product. This assumption is supported by the finding that thus far, no adverse reactions in dogs have been observed even after the repeated administration of rhTSH [5,6,9]. In selected dogs in which hypothyroidism had been suspected, we repeated the TSH stimulation test three times using rhTSH. Even though the third test was performed within 12 to 24 months after the first stimulation, no dogs had adverse reactions after the rhTSH administration [6].

The bTSH is commonly used also for *in vitro* experiments to test the influence of TSH [31-34]. With our data, we could show that other pituitary hormones, albumin, endotoxins and an extensive list of contaminating proteins (e.g., collagen, hemoglobin, superoxide dismutase, and cathepsin) are constituents in two different bTSH products. The effects of these impurities are rarely if ever considered in interpretations of the results from these cell culture experiments. However, in the light of our findings, the rhTSH should be preferred for *in vitro* usage if the sole effect of TSH has to be tested.

## Conclusion

The results of the present study show that rhTSH is a very pure product with minimal to no amounts of endotoxin, whereas bTSH is a crude mixture of proteins with a high level of endotoxin. Both, contaminating proteins like bovine albumin and the high endotoxin content in the bTSH can potentially lead to adverse reactions, which have been observed in the past, not only in humans, but also in veterinary medicine [10-14]. The highly variable and lot-dependent composition of the bTSH makes adverse reactions unpredictable. Consequently, although less expensive and easily available, bTSH cannot be regarded as an alternative to the more expensive rhTSH, and its use should be discouraged to prevent unintended side effects *in vitro* and *in vivo*.

## Methods

### Sample preparation

Two different bovine TSH products (Thyrotropic hormone from bovine pituitary; product 1 Sigma Aldrich, Buchs, Switzerland and product 2 Calbiochem, Merck Chemicals, Lucerne, Switzerland) and rhTSH (Thyrogen, Genzyme GmbH, Baar, Switzerland) were analyzed. All TSH products were dissolved in aqua ad iniectabilia (Fresenius Kabi AG, Stans, Switzerland), and 70 μg total protein of each TSH sample were loaded separately onto a 12%- Bis-Tris gel (NuPage Novex Bis-Tris Mini Gel, Invitrogen AG, Basel, Switzerland). After separation, the gels were stained with colloidal Coomassie Blue (RotiBlue, Roth, Karlsruhe, Germany) and the stained gel lanes were excised, digested in-gel, and finally dissolved in 3% acetonitrile/0.1% formic acid for MS analyses as previously described [35].

### Mass spectrometry analyses

Analyses were performed with an LTQ-FT-ICR Ultra mass spectrometer (Thermo Fisher Scientific, Pittsburgh, PA, USA) equipped with an Eksigent-Nano-high performance liquid chromatography system (Eksigent Technologies, Dublin, CA, USA) as described by Moretti et al. [35]. An inclusion list was used containing the m/z values of all doubly and triply charged tryptic peptides from human (P01222) and bovine (P01223) TSH.

### Peptide and protein identification (data analyses)

The MS data were analyzed and peptides identified with Mascot (Version 2.3.0, Matrix Science) using the Swiss-Prot database (release date, 05.Oct10; 521016 sequence entries). The peptide tolerance was set to ± 5 ppm, the MS/MS tolerance to ± 0.6 Da, and carbamidomethylation of cysteine was specified as a fixed modification.

### Determination of bovine growth hormone concentration

The concentration of bovine growth hormone (GH) was measured in duplicate by an Enzyme-linked Immunosorbant Assay (ELISA) using a GH antibody (10 mg/mL, 100 μL total volume, 1: 200 000 dilution, anti-ovine GH-3, Lot# AFP0802210Rb) obtained from Dr. Parlow [National Hormone & Peptide Program (NHPP) of the National Institute of Diabetes and Digestive and Kidney Diseases (NIDDK), National Institutes of Health, Bethesda, MD, USA]. The assay was performed as previously described, but with the following modifications [36,37]. The anti-ovine GH antibody was distributed into all wells of microtiter plates, which had been coated with anti-rabbit-globulin antiserum, and incubated for 24 h at room temperature. After decanting the supernatants, 15 μL of different concentrations of GH standard (0.8–100 ng/mL, NIDDK-bGH, AFP-10325C) diluted in assay buffer (7.1 g/L Na_2_HPO_4_, 1.1 g/L KH_2_PO_4_, 1.2 g/L NaCl, and 1.8 g/L EDTA, pH 7.5; containing 1% chicken serum; 100 μg/well), and samples were added and incubated for 24 h. After incubation, the supernatants were discarded and biotin-labeled GH was added and incubated for 3 h at room temperature. Finally, the substrate was added (40 min at 37°C), and after stopping, the reaction, the optical density was measured at 450 nm. Only concentrations in the linear range of the standard curve were evaluated. Therefore, lower and upper detection limit for undiluted samples were 2.0 and 50.0 ng/mL, respectively (working range). The EIA method was validated for protein extraction samples by analyzing linearity of dilution and intra-assay coefficient of variation (CV) using the respective TSH diluted in GH assay buffer. The signal linearity of dilution was 81–96%, the intra-assay CV was 15.1%, and the 50% binding of this assay system was 6.2 ng/mL. Protein samples were diluted 1:10 in water and additionally 1:20 in peptide buffer.

### Determination of bovine luteinizing hormone concentration

Bovine luteinizing hormone (LH) concentration was measured in duplicate using a solid phase two-site enzyme immunoassay kit (LH Detect, INRA, Tours, France). The immunoassay was validated for purified protein samples by analyzing linearity of dilution, and the intra-assay CV. The assay sensitivity was 1 ng/mL, and the intra-assay CV was 9.6%. Linearity of dilution was 82–102%. Protein for LH detection was diluted in water at 1:100 and additionally 1:100 in assay buffer. The standard curve ranged from 1.2 - 40 ng/mL.

### Endotoxin assay

Endotoxin levels were measured using a Limulus amebocyte lysate (LAL) kinetic chromogenic assay (Pyrogent LAL Chromogenic Assay, Lonza, Verviers, Belgium). The test is based on the measurement of the chromophore released from a suitable chromogenic peptide by the reaction of endotoxins with the lysate. Bacterial endotoxin was measured with upper and lower detection limits of 0.05 and 50 Endotoxin units (EU)/mL, respectively. The sample was mixed with the LAL/substrate reagent, placed in an incubating plate reader, and monitored over time for the appearance of a yellow color, according to the instructions of the manufacturer. The same TSH lots were used for endotoxin assays and mass spectrometry. Results are given in both EU/mL and EU/mg.

### Statistical analyses

Statistical support was provided by the biostatistical staff of the FGCZ. MS data were analyzed using Scaffold 3 (Proteome Software, Portland, OR, USA) The threshold for positive identification of a given protein was set to at least two peptides with a peptide probability of 95% or better. The minimal sequence coverage was set to a minimum of 10%. Data were further analyzed using non-parametric statistical methods (SPSS, Statistical Package for the Social Science, Software Packets for Windows, Version 18 and GraphPad PRISM® for Windows, Version 5.0) Kruskal-Wallis, Wilcoxon and Dunn’s multiple comparisons test were used. The Mann–Whitney-*U*-Test was used to determine differences between two groups (hormones, endotoxins). Values of p < 0.05 were considered statistically significant.

## Competing interests

None of the authors of this paper has a financial or personal relationship with other people or organizations that could inappropriately influence or bias the content of the paper. The authors declare that they have no competing interests.

## Authors’ contributions

SS carried out all the experimental work of the MS and drafted the manuscript. MP carried out the immunoassays and helped with revision of the manuscript. POH and BR coordinated and supervised the experimental work and helped with revision of the manuscript. BR did the biostatistical analyses. NSSR and CER advised on critical revision of the manuscript. FB designed, coordinated and supervised the study and wrote the manuscript. All authors read and approved the final manuscript.

## Supplementary Material

Additional file 1: Table S1 (Complete, including accession number). Complete list of the proteins identified in the bTSH product 1 (Thyrotropic hormone from bovine pituitary, Sigma Aldrich; 3 lot numbers) by database search following mass spectrometry. Numbers of assigned spectra are given and the minimal sequence coverage was set to a minimum of 10%. Contaminations like keratin or trypsin, which were registered in bTSH as well as in rhTSH were excluded from analyses. Click here for file

Additional file 2: Table S2 (Complete, including accession number). Complete list of identified proteins in the bTSH product 2 (TSH, bovine pituitary, Calbiochem Merck; 1 lot number) by database search following mass spectrometry. Numbers of assigned spectra are given and the minimal sequence coverage was set to a minimum of 10%. Contaminations like keratin or trypsin, which were registered in bTSH as well as in rhTSH were excluded from analyses. Click here for file

Additional file 3: Table S3 (Complete, including accession number). Complete list of identified proteins in the rhTSH preparation (Thyrogen, Genzyme GmbH; 2 lot numbers) by database search following mass spectrometry. Numbers of assigned spectra are given and the minimal sequence coverage was set to a minimum of 10%. Contaminations like keratin or trypsin, which were detected in bTSH as well as in rhTSH were excluded from analyses. Click here for file
