# Supplementary material for: Characterization of recombinant human and bovine thyroid-stimulating hormone preparations by mass spectrometry and determination of their endotoxin content
Source: BMC Vet Res. 2013 Jul 16;9:141. doi: 10.1186/1746-6148-9-141 (PMC3717043; doi:10.1186/1746-6148-9-141)
Supplement: Additional file 1: Table S1 — (Complete, including accession number). Complete list of the proteins identified in the bTSH product 1 (Thyrotropic hormone from bovine pituitary, Sigma Aldrich; 3 lot numbers) by database search following mass spectrometry. Numbers of assigned spectra are given and the minimal sequence coverage was set to a minimum of 10%. Contaminations like keratin or trypsin, which were registered in bTSH as well as in rhTSH were excluded from analyses. [file 1746-6148-9-141-S1.pdf]

Additional file 1

Table 1 (complete, including accession number)

Complete list of the proteins identified in the bTSH product 1 (Thyrotropic hormone from bovine pituitary, Sigma Aldrich; 3 lot numbers) by database search following mass spectrometry. Numbers of assigned spectra are given and the minimal sequence coverage was set to a minimum of 10%. Contaminations like keratin or trypsin, which were registered in bTSH as well as in rhTSH were excluded from analyses.

Identified Proteins	Accession Number	LOT 069K1588	LOT 119K1583	LOT 040M1246
Prolactin	sp|P01239|	143	105	60
Lutropin subunit beta	sp|P04651|	100	84	86
Vasopressin-neurophysin 2-copeptin	sp|P01180|	70	80	53
Thyrotropin subunit beta	sp|P01223|	70	54	44
Glycoprotein hormones alpha chain	sp|P01217|	57	73	75
Cathepsin D	sp|P80209|	46	38	48
Pro-opiomelanocortin	sp|P01190|	36	35	42
Stathmin	sp|Q3T0C7|	22	11	15
Serum albumin	sp|P49822|	17	0	0
Macrophage migration inhibitory factor	sp|P80177|	16	19	6
Somatotropin	sp|P01246|	12	11	8
Proteasome activator complex subunit 1	sp|Q4U5R3|	10	5	3
Prefoldin subunit 6	sp|Q17Q89|	9	0	6
Glucosylceramidase	sp|Q2KHZ8|	9	3	0
Cystatin-C	sp|P01035|	9	1	0
Selenium-binding protein 1	sp|Q2KJ32|	8	18	1
Vasopressin-neurophysin 2-copeptin	sp|P01185|	8	6	5
Cytochrome b-c1 complex subunit 7	sp|P00129|	8	4	3
Cathepsin H	sp|Q3T0I2|	8	2	3
Serum albumin	sp|P02769|	8	1	0
Ubiquitin	sp|P62990|	7	18	10
Prefoldin subunit 1	sp|Q3SZE2|	7	4	6
Ribonuclease UK114	sp|Q3T114|	7	4	4
Vasopressin-neurophysin 2-copeptin (Fragment)	sp|P01181|	7	1	3
Protein S100-A11	sp|P24480|	7	3	2
Cathepsin S	sp|P25326|	7	2	3
Lysozyme C, non-stomach isozyme	sp|P80189|	7	0	1
Thyrotropin subunit beta	sp|P01222|	6	10	0
Epididymal secretory protein E1	sp|P79345|	6	3	3
Fumarylacetoacetate hydrolase domain-containing protein 2	sp|Q2KIB0|	6	2	0
Phosphatidylethanolamine-binding protein 1	sp|P13696|	5	35	9

Connective tissue growth factor	sp|O18739|	5	1	5
Thioredoxin	sp|O97680|	5	1	0
Beta-2-glycoprotein 1	sp|P17690|	5	2	0
Palmitoyl-protein thioesterase 1	sp|P45478|	5	0	0
Acidic mammalian chitinase	sp|Q95M17|	5	0	0
Superoxide dismutase [Cu-Zn]	sp|P00442|	4	29	8
ATP synthase-coupling factor 6, mitochondrial	sp|P13618|	4	5	7
Hemoglobin subunit beta	sp|P02070|	4	2	3
Oxytocin-neurophysin 1	sp|P01175|	4	2	2
Cytochrome c oxidase subunit 5B, mitochondrial	sp|P00428|	4	2	0
Cytochrome c oxidase subunit 6B1	sp|P00429|	4	0	4
Insulin-like growth factor-binding protein 6	sp|Q05718|	4	0	2
Apolipoprotein A-I	sp|P02648|	4	0	0
Chromogranin-A	sp|P05059|	3	6	4
Mitochondrial import inner membrane translocase subunit Tim9	sp|Q2KIV2|	3	6	5
Proenkephalin-A	sp|P01211|	3	7	3
Coiled-coil domain-containing protein 58	sp|A4FUI1|	3	2	6
Mitochondrial import inner membrane translocase subunit Tim10	sp|Q2NKR1|	3	3	2
Beta-2-microglobulin	sp|P01888|	3	0	3
Spleen trypsin inhibitor I	sp|P04815|	3	0	2
Protein FAM136A	sp|Q2HJI3|	3	0	1
Transthyretin	sp|O46375|	3	0	0
Insulin-like growth factor-binding protein 7	sp|Q16270|	2	2	5
Glutathione peroxidase 1	sp|P00435|	2	0	7
Cathelicidin-4	sp|P33046|	2	2	1
Prefoldin subunit 5	sp|Q8HYI9|	2	2	1
COX assembly mitochondrial protein homolog	sp|Q3SZM6|	2	0	0
TP53-regulated inhibitor of apoptosis 1	sp|O43715|	1	4	2
Mitochondrial import inner membrane translocase subunit Tim13	sp|Q9Y5L4|	1	2	2
Acylphosphatase-1	sp|P41500|	1	6	0
Cytochrome c oxidase assembly protein COX19	sp|A8E4L1|	1	1	2
Coiled-coil-helix domain-containing protein 3, mitochondrial	sp|Q5E9D3|	0	2	3
Acyl-CoA-binding protein	sp|P07107|	0	2	0
