# Supplementary material for: Characterization of recombinant human and bovine thyroid-stimulating hormone preparations by mass spectrometry and determination of their endotoxin content
Source: BMC Vet Res. 2013 Jul 16;9:141. doi: 10.1186/1746-6148-9-141 (PMC3717043; doi:10.1186/1746-6148-9-141)
Supplement: Additional file 2: Table S2 — (Complete, including accession number). Complete list of identified proteins in the bTSH product 2 (TSH, bovine pituitary, Calbiochem Merck; 1 lot number) by database search following mass spectrometry. Numbers of assigned spectra are given and the minimal sequence coverage was set to a minimum of 10%. Contaminations like keratin or trypsin, which were registered in bTSH as well as in rhTSH were excluded from analyses. [file 1746-6148-9-141-S2.pdf]

Additional file 2

Table 2 (complete, including accession number)

Complete list of identified proteins in the bTSH product 2 (TSH, bovine pituitary, Calbiochem Merck; 1 lot number) by database search following mass spectrometry. Numbers of assigned spectra are given and the minimal sequence coverage was set to a minimum of 10%. Contaminations like keratin or trypsin, which were registered in bTSH as well as in rhTSH were excluded from analyses.

**Identified Proteins**	**Accession Number**	**LOT D00106386**
Beta-2-glycoprotein 1	sp|P17690|	52
Serum albumin	sp|P02769|	51
Lactotransferrin	sp|P24627|	45
Annexin A1	sp|P46193|	36
Annexin A6	sp|P79134|	35
Annexin A2	sp|A2SW69|	33
Hemoglobin subunit beta	sp|P02070|	32
Filamin-A	sp|P21333|	32
Phospholipase D3	sp|Q2KJJ8|	29
Stress-induced-phosphoprotein 1	sp|Q3ZBZ8|	25
Peptidyl-prolyl cis-trans isomerase B	sp|P80311|	25
Annexin A3	sp|Q3SWX7|	25
Eukaryotic translation initiation factor 5A-1	sp|P63241|	22
Gelsolin	sp|Q3SX14|	20
Spectrin alpha chain, brain	sp|P07751|	20
Metalloproteinase inhibitor 2	sp|Q9TRZ7|	20
Hepatoma-derived growth factor	sp|Q9XSK7|	20
Spectrin beta chain, brain	sp|Q01082|	19
Thrombospondin-1	sp|Q28178|	19
Hemopexin	sp|Q3SZV7|	17
Prolargin	sp|Q9GKN8|	17
Lutropin subunit beta	sp|P04651|	16
Adenylate kinase 2, mitochondrial	sp|P08166|	16
Glycylpeptide N-tetradecanoyltransferase 1	sp|O70310|	14
Interleukin enhancer-binding factor 3	sp|Q12906|	14
Rho GDP-dissociation inhibitor 1	sp|P19803|	13
Glycoprotein hormones alpha chain	sp|P01217|	13
Adenylyl cyclase-associated protein 1	sp|Q3SYV4|	13
Calcyclin-binding protein	sp|Q3T168|	13
Thyrotropin subunit beta	sp|P01223|	13
GTP:AMP phosphotransferase, mitochondrial	sp|P08760|	13
Hepatoma-derived growth factor-related protein 3	sp|Q923W4|	13

Vinculin	sp|P18206|	12
Transgelin	sp|Q9TS87|	12
Gamma-glutamyl hydrolase	sp|A7YWG4|	11
Neurofascin	sp|O94856|	11
Elongation factor 1-gamma	sp|Q3SZV3|	11
LDLR chaperone MESD	sp|Q3T0U1|	11
Glucosylceramidase	sp|Q2KHZ8|	10
Malate dehydrogenase, mitochondrial	sp|Q32LG3|	10
Peroxiredoxin-1	sp|Q5E947|	10
Vesicle-associated membrane protein-associated protein A	sp|Q0VCY1|	10
Neuroblast differentiation-associated protein AHNAK	sp|Q09666|	9
SUMO-conjugating enzyme UBC9	sp|P63279|	9
High mobility group protein B1	sp|A9RA84|	9
Phosphatidylinositol-binding clathrin assembly protein	sp|O55012|	9
Phosphatidylethanolamine-binding protein 1	sp|P13696|	9
Aminoacyl tRNA synthase complex-interacting multifunctional protein 1	sp|P31230|	9
GDNF family receptor alpha-1	sp|P56159|	8
Transcription elongation factor A protein 1	sp|Q29RL9|	8
Microtubule-associated protein RP/EB family member 1	sp|Q3ZBD9|	8
Peptidyl-glycine alpha-amidating monooxygenase	sp|P10731|	8
DNA-(apurinic or apyrimidinic site) lyase	sp|P23196|	8
Cathepsin L1	sp|P25975|	8
Protein canopy homolog 3	sp|Q0P5N1|	8
Serotransferrin	sp|Q29443|	8
Fermitin family homolog 2	sp|Q96AC1|	8
Gamma-aminobutyric acid receptor-associated protein-like 2	sp|P60519|	8
Ribosome-recycling factor, mitochondrial	sp|Q0VCQ4|	8
Cystatin-C	sp|P01035|	7
Methyl-CpG-binding protein 2	sp|P51608|	7
Myosin light chain kinase, smooth muscle	sp|Q28824|	7
Vasopressin-neurophysin 2-copeptin	sp|P01180|	7
Insulin-like growth factor-binding protein 7	sp|Q16270|	7
Tropomodulin-2	sp|Q9NZR1|	7
Tetranectin	sp|Q2KIS7|	7
D-tyrosyl-tRNA(Tyr) deacylase 1	sp|Q2T9V8|	7
BTB/POZ domain-containing protein KCTD12	sp|Q6WVG3|	7
Nascent polypeptide-associated complex subunit alpha, muscle-specific form	sp|P70670|	6
U2 small nuclear ribonucleoprotein A'	sp|P09661|	6
Complement factor B	sp|P81187|	6
Laminin subunit alpha-4	sp|Q16363|	6
Cochlin	sp|O43405|	6
Thioredoxin	sp|O97680|	6
78 kDa glucose-regulated protein	sp|P06761|	6

Plasminogen	sp|P06868|	6
RNA-binding protein FUS	sp|P35637|	6
Lumican	sp|Q05443|	6
Partner of Y14 and mago	sp|A6QPH1|	6
Somatotropin	sp|P01246|	6
Metalloproteinase inhibitor 1	sp|P20414|	6
Factor XIIa inhibitor	sp|P50448|	6
Ribosome maturation protein SBDS	sp|Q3SWZ6|	6
Glucosamine 6-phosphate N-acetyltransferase	sp|Q5RAL9|	6
Peroxiredoxin-2	sp|Q9BGI3|	6
Complement C3	sp|Q2UVX4|	5
Microtubule-associated proteins 1A/1B light chain 3 beta 2	sp|A6NCE7|	5
Sorbin and SH3 domain-containing protein 2	sp|O94875|	5
Glutaminyl-tRNA synthetase	sp|Q3MHH4|	5
Alpha-enolase	sp|Q9XSJ4|	5
Non-muscle caldesmon	sp|Q62736|	5
Elongation factor 1-alpha 1	sp|A2Q0Z0|	5
Microtubule-associated protein RP/EB family member 3	sp|Q5XIT1|	5
Clathrin coat assembly protein AP180	sp|O60641|	5
Heterogeneous nuclear ribonucleoprotein C	sp|O77768|	5
Heparin-binding growth factor 2	sp|P03969|	5
Haptoglobin	sp|Q2TBU0|	5
Calcium-dependent secretion activator 1	sp|Q9ULU8|	5
Eukaryotic translation initiation factor 3 subunit G	sp|O75821|	5
Nucleolin	sp|P19338|	5
Protein disulfide-isomerase A3	sp|P38657|	5
Ubiquitin carboxyl-terminal hydrolase 14	sp|P54578|	5
Coiled-coil-helix-coiled-coil-helix domain-containing protein 7	sp|Q17Q91|	5
Protein NipSnap homolog 3A	sp|Q5RAA9|	5
Protein dpy-30 homolog	sp|Q2NKU6|	5
Leiomodin-1	sp|Q8BVA4|	5
Spectrin beta chain, brain 1	sp|Q62261|	5
Heat shock cognate 71 kDa protein	sp|A2Q0Z1|	4
Protein AMBP	sp|P00978|	4
Lamina-associated polypeptide 2, isoform alpha	sp|P42166|	4
Transcription factor BTF3	sp|P20290|	4
Collagen alpha-1(XIV) chain	sp|Q80X19|	4
Pro-opiomelanocortin	sp|P01190|	4
Proteasomal ubiquitin receptor ADRM1	sp|A1L5A6|	4
Transcription activator BRG1	sp|A7Z019|	4
Collagen alpha-1(II) chain	sp|P02458|	4
Glia-derived nexin	sp|P07093|	4
40S ribosomal protein S19	sp|P17074|	4
60S ribosomal protein L12	sp|P23358|	4

40S ribosomal protein S3	sp|P23396|	4
Transgelin-2	sp|P37802|	4
Mesencephalic astrocyte-derived neurotrophic factor	sp|P80513|	4
Nuclear migration protein nudC	sp|Q17QG2|	4
Lysosomal alpha-mannosidase	sp|Q29451|	4
Complement C2	sp|Q3SYW2|	4
Acyl-CoA-binding domain-containing protein 7	sp|Q3SZF0|	4
Mitochondrial fission 1 protein	sp|Q3T0I5|	4
Tetratricopeptide repeat protein 1	sp|Q3ZBR5|	4
Ran-binding protein 3	sp|Q4R4T9|	4
Protein DJ-1	sp|Q5E946|	4
Talin-1	sp|Q9Y490|	4
Vesicle-associated membrane protein-associated protein B	sp|A2VDZ9|	4
Decorin	sp|P21793|	4
Myotrophin	sp|P58546|	4
Transcription factor BTF3 homolog 4	sp|Q2KIY7|	4
Cysteine and glycine-rich protein 1	sp|Q3MHY1|	4
Probable D-tyrosyl-tRNA(Tyr) deacylase 2	sp|Q96FN9|	4
Dynein light chain 1, axonemal	sp|Q2KID4|	4
DnaJ homolog subfamily A member 2	sp|O35824|	4
Beta-2-glycoprotein 1	sp|P33703|	4
Dynein light chain 2, cytoplasmic	sp|Q3MHR3|	4
40S ribosomal protein S3a	sp|B0KW94|	3
Ubiquitin-like protein 4A	sp|B2KIK3|	3
Synaptotagmin-1	sp|P21579|	3
Sulfhydryl oxidase 1	sp|O00391|	3
Pigment epithelium-derived factor	sp|Q95121|	3
Prelamin-A/C	sp|P02545|	3
Large proline-rich protein BAT3	sp|A5D9M6|	3
Heterogeneous nuclear ribonucleoprotein Q	sp|O60506|	3
Chromobox protein homolog 3	sp|Q13185|	3
Protein S100-A13	sp|P79342|	3
Collagen alpha-1(XVIII) chain	sp|P39061|	3
Inter-alpha-trypsin inhibitor heavy chain H5	sp|A2VE29|	3
Agrin	sp|O00468|	3
Hemoglobin subunit alpha	sp|P01966|	3
Cofilin-1	sp|P10668|	3
Pleiotrophin	sp|P21782|	3
Catenin alpha-1	sp|P26231|	3
Annexin A11	sp|P27214|	3
Cholinesterase	sp|P32749|	3
Antithrombin-III	sp|P41361|	3
Arylsulfatase K	sp|Q148F3|	3
Poly(rC)-binding protein 2	sp|Q15366|	3

High mobility group protein B3	sp|Q32L31|	3
PDZ and LIM domain protein 7	sp|Q3SX40|	3
Leucine zipper transcription factor-like protein 1	sp|Q3ZBL4|	3
Transcobalamin-2	sp|Q9XSC9|	3
40S ribosomal protein S20	sp|A1XQU9|	3
Follitropin subunit beta	sp|P04837|	3
Thrombospondin-1	sp|P07996|	3
Microtubule-associated protein 4	sp|P27546|	3
Gamma-aminobutyric acid receptor-associated protein-like 1	sp|P60518|	3
Retinoic acid receptor responder protein 2	sp|Q29RS5|	3
PDZ and LIM domain protein 4	sp|Q3T005|	3
Serine/arginine repetitive matrix protein 1	sp|Q5ZMJ9|	3
Insulin-like growth factor-binding protein 7	sp|Q61581|	3
Aspartyl-tRNA synthetase, cytoplasmic	sp|Q3SYZ4|	2
Heterochromatin protein 1-binding protein 3	sp|Q08DU9|	2
Epsin-2	sp|O95208|	2
Insulin-like growth factor-binding protein 3	sp|P20959|	2
PC4 and SFRS1-interacting protein	sp|Q8MJG1|	2
THO complex subunit 4	sp|B5FXN8|	2
Vacuolar protein sorting-associated protein 4B	sp|O75351|	2
Ubiquitin-conjugating enzyme E2-17 kDa	sp|P25867|	2
Endoplasmic reticulum resident protein 29	sp|P30040|	2
Activated RNA polymerase II transcriptional coactivator p15	sp|P53999|	2
Tissue factor pathway inhibitor 2	sp|Q7YRQ8|	2
Glycogen phosphorylase, liver form	sp|Q0VCM4|	2
Protein S100-A10	sp|P04163|	2
Cystatin-B	sp|P25417|	2
Small nuclear ribonucleoprotein Sm D3	sp|P62318|	2
Ribosomal L1 domain-containing protein 1	sp|A4FV97|	2
Transforming growth factor-beta-induced protein ig-h3	sp|O11780|	2
NSFL1 cofactor p47	sp|O35987|	2
PDZ and LIM domain protein 3	sp|O70209|	2
Epsin-1	sp|O88339|	2
Kinectin	sp|O97961|	2
Histone H1.4 (Fragment)	sp|P02252|	2
Collagen alpha-2(I) chain	sp|P02465|	2
Fibronectin	sp|P02751|	2
Sodium/potassium-transport. ATPase subunit beta-1	sp|P05028|	2
Collagen alpha-3(VI) chain	sp|P12111|	2
ATP synthase subunit O, mitochondrial	sp|P13621|	2
Lamin-B1	sp|P14731|	2
Oxysterol-binding protein 1	sp|P16258|	2
Mimecan	sp|P19879|	2
Secretogranin-1	sp|P23389|	2

Tenascin	sp|P24821|	2
Peptidyl-prolyl cis-trans isomerase FKBP2	sp|P26885|	2
Elongation factor 1-beta	sp|P34826|	2
Protein S100-A4	sp|P35466|	2
60S ribosomal protein L30	sp|P58372|	2
DnaJ homolog subfamily B member 11	sp|P81999|	2
Eukaryotic translation initiation factor 3 subunit J	sp|Q0VCU8|	2
Ubiquitin-conjugating enzyme E2 variant 1	sp|Q13404|	2
ELAV-like protein 1	sp|Q15717|	2
CB1 cannabinoid receptor-interacting protein 1	sp|Q17QM9|	2
Heat shock 70 kDa protein 1B	sp|Q27965|	2
Collagen alpha-1(XII) chain (Fragment)	sp|Q28902|	2
Suppressor of G2 allele of SKP1 homolog	sp|Q2KIK0|	2
Vitamin D-binding protein	sp|Q3MHN5|	2
Hepatoma-derived growth factor-related protein 2	sp|Q3UMU9|	2
PDZ and LIM domain protein 5	sp|Q96HC4|	2
Cadherin EGF LAG seven-pass G-type receptor 2	sp|Q9HCU4|	2
