# Supplementary material for: Characterization of recombinant human and bovine thyroid-stimulating hormone preparations by mass spectrometry and determination of their endotoxin content
Source: BMC Vet Res. 2013 Jul 16;9:141. doi: 10.1186/1746-6148-9-141 (PMC3717043; doi:10.1186/1746-6148-9-141)
Supplement: Additional file 3: Table S3 — (Complete, including accession number). Complete list of identified proteins in the rhTSH preparation (Thyrogen, Genzyme GmbH; 2 lot numbers) by database search following mass spectrometry. Numbers of assigned spectra are given and the minimal sequence coverage was set to a minimum of 10%. Contaminations like keratin or trypsin, which were detected in bTSH as well as in rhTSH were excluded from analyses. [file 1746-6148-9-141-S3.pdf]

Additional file 3

Table 3 (complete, including accession number)

Complete list of identified proteins in the rhTSH preparation (Thyrogen, Genzyme GmbH; 2 lot numbers) by database search following mass spectrometry. Numbers of assigned spectra are given and the minimal sequence coverage was set to a minimum of 10%. Contaminations like keratin or trypsin, which were detected in bTSH as well as in rhTSH were excluded from analyses.

**Identified Proteins**	**Accession Number**	**LOT A8035H40**	**LOT A8063H19**
Thyrotropin subunit beta	sp|P01222|	116	126
Glycoprotein hormones alpha chain	sp|P01215|	25	26
Glyceraldehyde-3-phosphate dehydrogenase	sp|P04406|	6	1
Calmodulin-like protein 5	sp|Q9NZT1|	5	1
Caspase-14	sp|P31944|	4	1
Serpin B12	sp|Q96P63|	5	0
Galectin-7	sp|P47929|	2	0
